# Interventions for Workplace Violence Prevention in Emergency Departments: A Systematic Review

**DOI:** 10.3390/ijerph18168459

**Published:** 2021-08-10

**Authors:** Tanja Wirth, Claudia Peters, Albert Nienhaus, Anja Schablon

**Affiliations:** 1 Institute for Occupational and Maritime Medicine (ZfAM), University Medical Centre Hamburg-Eppendorf (UKE), 20459 Hamburg, Germany; 2 Competence Centre for Epidemiology and Health Services Research for Healthcare Professionals (CVcare), Institute for Health Services Research in Dermatology and Nursing (IVDP), University Medical Centre Hamburg-Eppendorf (UKE), 20246 Hamburg, Germany; c.peters@uke.de (C.P.); a.nienhaus@uke.de (A.N.); a.schablon@uke.de (A.S.); 3 Department for Occupational Medicine, Hazardous Substances and Health Sciences (AGG), German Social Accident Insurance for the Health and Welfare Services (BGW), 22089 Hamburg, Germany

**Keywords:** emergency service, hospital, health personnel, workplace violence, prevention, occupational health, systematic review

## Abstract

Emergency departments (EDs) are high-risk settings for workplace violence, but interventions to prevent violent incidents and to prepare staff are not yet consistently implemented, and their effectiveness is often unclear. This study aims to summarise evidence on workplace violence prevention interventions that were implemented in EDs to reduce violent incidents caused by patients/relatives or to increase the knowledge, skills or feelings of safety of ED staff. A systematic review was conducted. The databases MEDLINE, Web of Science, Cochrane Library, CINAHL and PsycINFO were searched for studies dated between January 2010 and May 2021. Interventional and observational studies reporting on behavioural, organisational or environmental interventions among healthcare workers in hospital EDs were included. Studies were assessed for methodological quality using the Johanna Briggs Institute Tools. Key findings of studies were summarised narratively. Fifteen studies were included, of which eleven examined behavioural interventions (classroom, online or hybrid training programmes) on de-escalation skills, violent person management or self-defence techniques. Four studies included in addition, organisational and environmental interventions. Most studies showed that interventions had a positive effect in the form of a reduction of violent incidents or an improvement in how prepared staff were to deal with violent situations; however, evidence is still sparse. Further studies should consider in particular, environmental and organisational interventions and ensure a high methodological quality.

## 1. Introduction

Workplace violence in healthcare is a global and highly prevalent problem; within the healthcare sector, emergency departments (EDs) are considered a high-risk setting for workplace violence [1,2]. Workplace violence has been defined by the International Labour Organisation (ILO) [3] (p. 4) as “any action, incident or behaviour that departs from reasonable conduct in which a person is assaulted, threatened, harmed, injured in the course of, or as a direct result of, his or her work”. Accordingly, workplace violence can be of a physical or psychological nature [4,5]. Workplace violence can be categorised into four types based on its source: criminal intent, customer/client, worker-on-worker, and personal relationship. In the healthcare sector, violence perpetrated by customers or clients (in this case patients) is most common [6]. Liu et al. [1] examined global prevalence rates of workplace violence caused by patients and visitors against healthcare workers in a meta-analysis. The global 12-month prevalence in EDs was 31% (95% CI, 26%–36%) for physical violence and 62.3% (95% CI, 53.7%–70.8%) for nonphysical violence. Among employees in EDs of a large German hospital, the most frequent forms of reported physical violence incidents (n = 2853) were holding/clinging (22%), pinching (17%) and spitting (16%). For nonphysical violence incidents (n = 15,126) these were grumbling (40%), shouting (19%) and insulting (19%) [7]. In emergency primary healthcare clinics in Norway, 60% of 320 aggressive incidents were considered as severe (score of ≥ 9 on a scale from 0 to 21) [8]. In EDs, several factors converge that can contribute to the occurrence of violent incidents. On the part of the patients, literature reviews describe alcohol and drug intoxication and mental illnesses as important risk factors [9,10,11]. Concerning the organisation and staff in EDs, night shifts [10,12], long waiting times for patients, high job demands of staff [11,12] and an inadequate worker–patient relationship [11], increase the risk of workplace violence. Experiences of workplace violence can have serious negative consequences for ED staff. Italian ED healthcare workers who had suffered incidents of violence reported effects on lifestyle, such as sleep disorders and changes from social relationships to social isolation [13]. About 20% of 252 reported physical assaults in three EDs in the USA resulted in an injury [14]. ED staff who had suffered verbal abuse reported consequences on their mental health and well-being, such as irritation, anger, depression, anxiety, guilt, humiliation, feelings of helplessness and disappointment [11]. In addition, exposure to nonphysical violence can significantly impact symptoms of burnout, secondary traumatic stress and compassion satisfaction in ED staff [15]. Negative consequences can also affect the organisation. The experience of workplace violence among ED nurses in the USA was related to experiences of negative stress, decreased work productivity, and the quality of patient care [16]. Therefore, prevention of workplace violence is of great importance, but measures are not yet consistently implemented [17]. In a survey of nursing staff (n = 105) in EDs of seven German hospitals, 73% of the participants stated that they did not feel safe at their workplace and that they were not well prepared for incidents of violence [18].

Prevention interventions can be categorised into prevention, protection and treatment approaches. While treatment approaches aim to reduce the negative impact of violent incidents, prevention and protection approaches proactively aim to reduce the risk of violence or improve the handling of violent incidents [4]. The latter two can be implemented at an environmental, organisational and/or behavioural level. According to guidelines on the prevention of workplace violence in the healthcare sector, environmental changes could be implemented in the form of controlled access, good lighting, clear signs, comfortable waiting areas, alarm systems, surveillance cameras and the removal or securing of weaponisable furniture. At an organisational level, it is further recommended to ensure that staffing is sufficient and adequate, to avoid having staff work alone, to circulate information on patients, to practice open communication, and to improve work practices. Finally, interventions possible at a behavioural level include training of staff members, superiors and managers on policies and procedures, de-escalation and self-defence techniques [4,5,19]. However, the feasibility and effectiveness of interventions for prevention in EDs, e.g., in terms of how they reduce violent incidents and improve the knowledge of ED staff, and help them to feel safe and at ease, are still unclear [12,18]. This is why, recent literature has been systematically reviewed and summarised.

This systematic review aims to summarise the existing evidence from evaluation studies on the prevention of patient-on-employee violence and aggression in EDs, where the purpose of the studies was to reduce the frequency of violent incidents, to increase knowledge, skills, or awareness related to violent incidents, or to help ED staff feel safer and more at ease.

## 2. Materials and Methods

The conduct and description of this systematic review follows the Preferred Reporting Items for Systematic Reviews and Meta-Analyses (PRISMA) [20]. Prior to conducting the review, a detailed study protocol was prepared on the procedures and methods planned. The protocol is available in German language and can be obtained from the corresponding author on request.

### 2.1. Eligibility Criteria

The screening and selection of studies was based on predefined inclusion and exclusion criteria according to the PIO scheme (population, intervention, outcome), supplemented by criteria for exposure and study design and by specified report characteristics (publication type, date, study region). Table 1 provides an overview of the eligibility criteria. The presence of a control group was not a requirement for inclusion in this review. We included studies that had an external control group, a pre/post design, where participants were their own controls, and also studies that had no controls. Moreover, no studies were excluded on the basis of language.

### 2.2. Search Strategy

The literature search was conducted in the electronic databases MEDLINE (via PubMed), Web of Science, Cochrane Library, CINAHL and PsycINFO on 14 September 2020. An update of the search was conducted on 31 May 2021. Databases were searched for entries dating from the year 2010 onwards. The search string combined keywords concerning the population/occupation (e.g., “health personnel”), the population/setting (e.g., “emergency service*”), the exposure (e.g., “aggression*”) and the intervention (e.g., “education*”). The search string was first developed for MEDLINE and then adapted to the other databases (Supplementary Table S1). Additionally, reference lists of included studies and reviews on similar topics were hand-searched for further relevant studies.

### 2.3. Study Selection

All records identified through the literature search were transferred to the literature management program EndNote and duplicates were removed. The screening of titles and abstracts for relevant studies was conducted by one reviewer (TW). Unclear titles/abstracts were screened by another reviewer (CP) and discussed by both reviewers until consent for inclusion or exclusion was achieved. Full-text articles were screened independently by two reviewers (TW and CP) using a standardised screening instrument, including the eligibility criteria for study design, publication type, study region, study population, exposure, intervention and outcome. Studies that met all criteria were included in the review. Whenever the two reviewers came to differing conclusions about inclusion or exclusion, these were resolved by discussion.

### 2.4. Data Extraction

Firstly, the characteristics of included studies were extracted by a reviewer (TW) using a standardised data form and verified by a second reviewer (CP). The extracted information included first author, publication date, study region, study design, study setting and population, intervention description, follow-up period, and primary and secondary outcome parameters. Secondly, key findings of the included studies were extracted by one reviewer (TW) using a standardised Microsoft^®^ Excel^®^ spreadsheet. A second reviewer (CP) verified the accuracy of the extracted data.

### 2.5. Quality Assessment

A quality assessment of the included studies was performed using the Critical Appraisal Tools of the Johanna Briggs Institute (JBI) [21,22]. The Checklist for Quasi-Experimental Studies was used for most of the included studies [22]. It comprised nine items with the four possible response categories being “yes”, “no”, “unclear” and “not applicable”. Item eight, originally asking for outcomes being measured in a reliable way, was not applicable to most studies. Therefore, it was slightly adjusted to determine whether validated instruments were used. Three descriptive cross-sectional studies were appraised using the Checklist for Prevalence Studies [21]. It also comprised nine items with the same four response categories. Two appraisers independently assessed the quality of the studies (TW and CP). Differing conclusions were resolved by discussion. An overall score for each study was calculated by summing the number of “yes” responses. A score of ≤3 was considered as low quality, from 4 to 6 as moderate and ≥7 as high quality. No studies were excluded on the basis of methodological quality.

### 2.6. Synthesis of Results

The main characteristics of the included studies were analysed descriptively and summarised in a table. A narrative summary of the key findings was provided. It included a description of the different types of interventions and results on the main outcomes. Differences and similarities between studies were highlighted and methodological quality was considered. It was not possible to perform pooled analyses (meta-analyses) due to the large heterogeneity of the studies in terms of the interventions performed, survey instruments used and outcome parameters investigated.

## 3. Results

### 3.1. Study Selection

**Figure 1:**
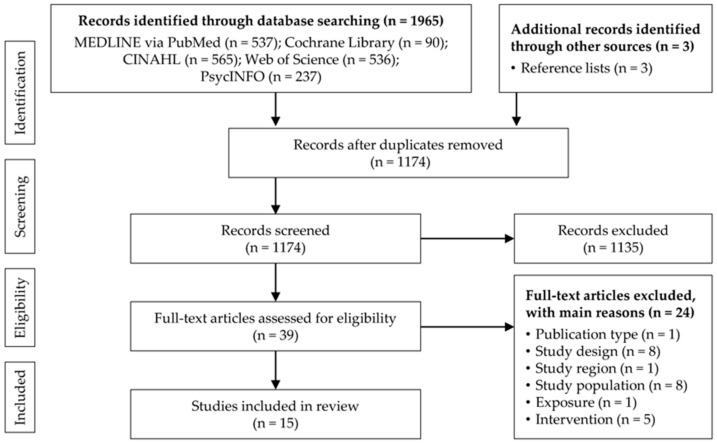
Flow chart of the study selection process.

Overall, 1965 records were identified through the database search and three additional studies through the screening of reference lists. After duplicates were removed, 1174 titles/abstracts were screened and subsequently, 39 full-text articles were assessed for eligibility. Fifteen studies were included in the review (Figure 1).

### 3.2. Study Characteristics

Table 2 provides an overview of the characteristics of the included studies. Of the fifteen studies, ten were conducted in the USA [14,23,24,25,26,27,28,29,30,31], two in Australia [32,33], two in France [34,35] and one in Germany [36]. Five studies were published in each of the three time periods, from 2010 to 2013 [26,27,32,33,34], from 2014 to 2017 [14,23,25,28,31], and from 2018 to 2021 [24,29,30,35,36], respectively. Ten studies had a quasi-experimental design, most of them using pre- and post-tests [14,23,24,26,28,29,30,31,33] and one identifying itself as an interrupted time-series study [35]. One was a mixed-methods study with its main component being a pre- and post-test survey [32]. One study described itself as an observational study [25] and three were cross-sectional evaluation studies [27,34,36]. Eleven studies implemented behavioural interventions [23,24,25,26,28,29,31,32,33,34,36]. Four studies used multi-component approaches including behavioural, organisational and environmental interventions [14,27,30,35]. As an outcome measure, three studies examined knowledge attainment of participants through a test score [23,26,28], eight studies included self-reported knowledge, confidence, ability, skills or attitudes of staff [23,24,29,30,31,32,33,36], four studies measured a change in violence incidence [14,25,30,35] and two studies merely evaluated the satisfaction with or success of the intervention [27,34].

### 3.3. Quality Assessment

Three studies were classified as being of low [27,30,34], ten of moderate [23,24,25,26,28,29,31,33,35,36] and two of high quality [14,32] (see quality score in Table 2). Quasi-experimental studies most often lacked an independent control group, multiple pre/post measurements, a complete follow-up, an adequate description of those lost to follow-up or valid outcome measures. For the cross-sectional studies, a detailed description of the study subjects and setting, sufficient coverage of the identified sample, or the use of valid methods for the identification of the condition, were most often not provided. Further detailed critical appraisal results are provided in the Supplementary Materials (Tables S2 and S3).

**Table 2:** Characteristics of the included studies (n = 15).

Reference, Country	Study Design	Setting	Study Size (n), Population, Sex, Age	Comparison Group	Intervention	Follow-up Period	Related Outcome Measures	Quality Score
Ball et al. (2015) [23], USA	Pre- and post-test study	Suburban academic level I trauma centre	93 fourth-year medical students during their 4-week ED clerkship, 58.1% female, mean age: 26.8 years	Matched pre- and post-surveys; 30 students who did not watch video	10-min video podcast covering learning objectives in violent person management	Pre-test: during the 4-week ED clerkship Post-test: at the final examination	Knowledge attainment (change in test score), change in self-reported confidence in identifying and responding to a violent situation	6/9
Bataille et al. (2013) [34], France	Cross-sectional, single centre evaluation study	Emergency intensive care unit of the general hospital of Narbonne	27 medical and paramedical employees, sex and age NR	N/A	Training with the main objective of defusing a conflict situation (basics of conflict psychology, self-defence gestures and postures)	N/A	Satisfaction with the training	2/9
Buterakos et al. (2020) [24], USA	Quasi-experimental study with two phases	ED (level I trauma centre for adults and level II trauma centre for paediatrics) in an urban hospital	Phase I: 25 nurses, 72% female, 40% 31–40 years Phase II: 34 nurses, 76.5% female, age NR	Matched pre- and post-surveys	5-min educational in-service training sessions and reinforcement posters on: phase I: importance of reporting; phase II: assertive de-escalation and self-protection	Phase I: baseline and 1-month post-intervention Phase II: baseline and 2-month post-intervention	Increase in the reporting of assaults, increase in nurses’ confidence in de-escalation and ability to protect themselves during assaults	4/9
Frick et al. (2018) [36], Germany	Cross-sectional evaluation study	Acute care units (EDs, paediatric EDs and obstetrics) at the Charité Berlin	110 staff members (92.3% nurses), sex and age NR	N/A	Three 8-h days of in-house de-escalation training by multipliers	N/A	Self-assessment and application of skills after the training (detection of warning signals, verbal de-escalation, defence and escape techniques, dealing with provocative behaviour)	4/9
Gerdtz et al. (2013) [32], Australia	Mixed methods, multisite evaluation study (pre- and post-test survey and individual interviews)	Public-sector EDs in Victoria	Survey: 471 registered nurses and midwives, 86.6% female, 33.1% 20–29 years Interviews: 28 nurse unit managers and trainers, 85.7% female, age NR	Matched pre- and post-surveys	Management of Clinical Aggression–Rapid Emergency Department Intervention (MOCA-REDI) programme (45 min. in-service session, train-the-trainer model)	Survey: before and 6–8 weeks after training Interviews: 8–10 weeks after training	Survey: staff attitudes about the causes and management of patient aggression Interviews: staff perceptions of the impact of the training	7/9
Gillam (2014) [25], USA	Single-phase observational study	Primary ED of an acute tertiary care hospital	ED staff (n, sex and age NR)	Monthly code purple activity	8-h nonviolent crisis intervention training programme for ED staff	November 2012 to October 2013	Change in code purple incidence (violent events that initiate emergency response by hospital security team) in terms of completed training	5/9
Gillespie et al. (2012) [26], USA	Quasi-experimental study	Three EDs (one level I trauma centre, one urban ED, one suburban ED) in the Midwestern USA	315 employees from the EDs (47.9% unlicensed assistive personnel), sex and age NR	Matched pre- and post-surveys; comparison: web-based learning only (n = 95) vs. hybrid group (n = 220)	Educational programme: web-based learning programme (units 1–3) and web-based/classroom-based hybrid learning programme (units 1–3 and unit 4)	Pre-test: prior to unit 1 Post-test: following completion of the programme with or without unit 4	Knowledge attainment (change in test score)	4/9
Gillespie et al. (2013) [27], USA	Cross-sectional evaluation study using action research	Three EDs (one level I trauma centre, one urban ED, one suburban ED) in the Midwest USA	53 ED employees (66% nurses), sex and age NR	N/A	(1) Walk-throughs with recommendation of environmental changes (2) policies and procedures for each hospital (3) online and classroom training	N/A	ED employees’ rating of the programme’s benefit, ease of implementation, level of commitment and importance of (sub)components	1/9
Gillespie et al. (2014) [28], USA	Quasi-experimental, repeated measures study	Two paediatric EDs (one community based, one level I trauma centre) and one adult/paediatric ED (university-affiliated level I trauma centre), Midwest USA	120 employees (71.7% registered nurses), 86.7% female, age NR	Matched pre- and post-surveys	Hybrid workplace violence educational programme with online and classroom components	Time 1: prior to online modules Time 2: after completing online modules Time 3: 6 months after classroom module	Knowledge attainment and retention on preventing, managing, and reporting incidents of workplace violence (change in test score)	6/9
Gillespie et al. (2014) [14], USA	Quasi-experimental, repeated measures study	Three EDs (one level I trauma centre, one urban tertiary care ED, one community-based suburban ED)	209 ED employees (56% nurses), 71.3% female, mean age: 37.3 years	Three comparison site EDs	(1) Walk-throughs with recommendation of environmental changes (2) policies and procedures for each hospital (3) online and classroom training	Monthly survey for 9 months before the intervention and 9 months after the intervention	Reduction of the incidence of physical assaults and threats against ED employees by patients and visitors	8/9
Hills et al. (2010) [33], Australia	Pre- and post-test study	Rural hospital EDs and health services in New South Wales	55 (pre-survey)/33 (post-survey) ED and Mental Health Service clinicians and Health and Security Assistants, sex and age NR	Unmatched pre- and post-surveys	24-week online learning programme including i.a.: assessing, identifying and managing risk and safety, therapeutic communication and de-escalation skills	Survey: before and after completing the programme	Knowledge and skill development (perceived self-efficacy and confidence in dealing with aggressive behaviour and mental health issues)	4/9
Krull et al. (2019) [29], USA	Pre- and post-test study	ED in the Upper Midwest region of the USA	96 interprofessional ED staff (55% registered nurses), 74% female, age NR	Matched pre- and post-surveys	Individual computer-based and simulation training (20-min patient scenario, 25-min debriefing session) on de-escalation techniques and restraint application	Pre- and post-survey directly before and after the simulation training	Knowledge, skills, abilities, confidence, and preparedness to manage aggressive or violent patient behaviour	6/9
Okundolor et al. (2021) [30], USA	Pre- and post-test study and retrospective review of incident report system	Psychiatric ER of the ED of a large, urban, public, academic hospital in Los Angeles	42 psychiatric ER nursing staff, sex and age NR	Matched pre- and post-surveys and monthly incidents	(1) behavioural response team drills (2) pre-shift briefing (3) screening for patients’ risk for violence (4) posting signage (5) countermeasure interventions (6) post-assault debriefing (7) post-assault support	Survey: before, during and after the interventions Record review: monthly from May 2016 to September 2018	Perceived self-efficacy in managing patients with a propensity for violence, number of physical assaults (with harm scores ≥5) on staff per month	3/9
Touzet et al. (2019) [35], France	Single-centre, prospective interrupted time-series study	Adult ophthalmology ED of an urban university hospital in the Rhône-Alpes region of France	30 healthcare workers (23% nurses, 23% residents), sex and age NR	Pre–post analysis	(1) computerised triage algorithm (2) signage (3) messages broadcast in waiting rooms (4) mediator (5) video surveillance	3-month pre-interventional period, 3-month training period and 12-month implementation period of the programme	Violent acts committed by patients or persons accompanying them against healthcare workers, other patients or persons accompanying patients among all admissions	5/9
Wong et al. (2015) [31], USA	Pre- and post-test study	ED	106 ED staff members (41% nurses), 58% female, 34% 26–30 years	Matched pre- and post-surveys	Simulation-enhanced interprofessional curriculum (30-min lecture, two simulation scenarios, structured debriefing)	Pre- and post-survey directly before and after the course	Staff attitudes towards management of patient aggression	6/9

Abbreviations: ED = emergency department, ER = Emergency Room, N/A = not applicable, NR = not reported.

### 3.4. Results on Behavioural Interventions

#### 3.4.1. Online Training Programmes

Two studies implemented online training and measured the effect on staff knowledge, skills and confidence in identifying and dealing with violent situations. Ball et al. [23] provided a 10-min video podcast on violent person management to fourth-year medical students during their emergency medicine clerkship. They found a significant improvement in knowledge test scores after watching the podcast (mean difference 1.77, 95% CI 1.42–2.13, *p* < 0.001, n = 93) and higher test scores compared to 30 students who did not watch the podcast (mean 5.82 ± 1.38 vs. 4.13 ± 1.48, *p* < 0.001). In addition, the proportion of students feeling confident in responding to a violent person significantly increased (15.0% vs. 47.3%, *p* = 0.00) but not the proportion of those feeling confident in identifying a potentially violent person (48.4% vs. 80.9%, *p* = 0.21).

Hills et al. [33] implemented a 24-week online learning programme, e.g., on risk and safety and de-escalation skills, in general hospital EDs and Mental Health Services, and found a significant improvement in perceived self-efficacy in dealing with aggressive behaviours of clients (mean 21.5 ± 6.0, n = 55 vs. 26.3 ± 3.9, n = 33, *p* < 0.001). A further significant improvement was seen in confidence for deciding if a person might be at risk of harming others (mean 4.5 ± 1.2 vs. 5.6 ± 0.8, *p* < 0.001).

#### 3.4.2. Classroom Training Programmes

Six studies reported on interactive classroom or short in-service training sessions, which all included components on de-escalation and/or self-defence techniques. Buterakos et al. [24] used short in-service sessions and reinforcement posters on reporting, de-escalation and self-protection. They found no significant difference in the confidence of 34 participants regarding de-escalating an aggressive patient before and after the intervention (Z = −1.022, *p* = 0.27). Gerdtz et al. [32] also implemented an in-service training session via a train-the-trainer model on de-escalation techniques and effective communication skills for the prevention of patient aggression. They found only limited evidence (statistically significant changes only in one fifth of their items tested) for changes in attitudes about the causes and management of aggression. Interviewed managers/trainers did, however, perceive a positive impact on the way staff worked to prevent patient aggression. Gillam [25] examined the effect of an 8-h nonviolent crisis intervention training session on the incidence of violence in one hospital ED. The training included techniques to de-escalate potentially violent situations and to avoid injuries. The author found a significant decrease in violent events that initiated emergency responses by the hospital security team when more staff were trained in the previous 90–150 days, but not when the time of training was further back. Wong et al. [31] developed an interprofessional curriculum including case-based simulations that incorporated de-escalation and self-defence techniques, team-based approaches, the application of physical restraints and medication. Participants’ attitudes towards patient aggression factors significantly improved from pre- to post-intervention (all *p* < 0.01), except for the clinical management of aggression (*p* = 0.54). The other two studies did not provide a comparison over time. Bataille et al. [34] implemented training with the main objective of defusing a conflict situation. The training was satisfactory and clear to all 27 participants, with 96% feeling that they had learned new things; all wanted to continue and expand the training. Frick et al. [36] evaluated a three-day in-house de-escalation training programme using multipliers. After the training, skills were rated as “very high” or “high” by 56.5% for the detection of warning signals, by 48.1% for verbal de-escalation, by 25.2% for defence/escape techniques and by 44.4% for dealing with provocative behaviour (of n = 110).

#### 3.4.3. Hybrid Training Programmes

Three studies examined the effects of hybrid educational interventions on ED staff knowledge on preventing and managing workplace violence. In two studies, Gillespie et al. [26,28] implemented an educational programme consisting of web-based and classroom-based learning units. There was a significant increase in knowledge among participants of the web-based unit (t = 5.008, *p* < 0.001, n = 95) and also among participants of the web-based and classroom-based unit (t = 9.629, *p* < 0.001, n = 220) compared to scores before the intervention. The knowledge attainment did not differ significantly between both groups [26]. Gilliespie et al. [28] still found a significant increase in knowledge on preventing, managing and reporting incidents of workplace violence at six months after completion of the full programme (F = 53.454, *p* < 0.001). Krull et al. [29] provided computer-based training followed by simulation training on de-escalation techniques and restraint application. ED staff perceived their knowledge, skills, ability, confidence and preparedness to manage aggressive patient behaviour to be significantly higher after the training (all *p* < 0.001).

### 3.5. Results on Multicomponent Interventions

Four studies used multicomponent programmes, including behavioural, organisational and environmental interventions, to reduce the number of verbal violent events, threats and/or physical assaults against ED staff. In two studies, Gillespie et al. [14,27] examined the effects of a programme that included walk-throughs to identify environmental risks and implement site-specific changes, the formulation of best practice policies and procedures, and online and classroom training for workplace violence prevention and management. The programme was rated by employees (n = 53) as moderately beneficial. The programme subcomponents seen as most important were surveillance and monitoring, environmental changes and classroom education [27]. In addition, there was a significant decrease in the incidence of physical assaults and threats against ED workers in the intervention EDs from pre- to post-intervention. However, this was also observed for the comparison group without the intervention [14]. Okundolor et al. [30] examined the effect on the number of physical assaults on staff, of a multifaceted intervention including behavioural response team drills, pre-shift briefing, screening for patients’ risk for violence, the posting of signage, countermeasure interventions and post-assault debriefing and support. They observed a 75% decrease of assaults between July 2017 and June 2018, as compared to the same time period in the previous year. Touzet et al. [35] implemented a programme including a computerised triage algorithm, signage, messages broadcast in waiting rooms, the presence of a mediator and video surveillance. The number of self-reported acts of violence significantly decreased from 24.8 per 1000 admissions (95% CI 20.0–29.5) preintervention, to 9.5 per 1000 (95% CI 8.0–10.9) in the intervention period (*p* < 0.001).

## 4. Discussion

This systematic review summarises the current research on workplace violence prevention interventions aiming to reduce the frequency of violent incidents in EDs or increase ED staff knowledge, skills or confidence to manage aggressive patient behaviour. A total of 15 studies published since the year 2010 were identified. Eleven of them examined behavioural interventions in the form of classroom, online or hybrid training programmes on de-escalation skills, violent person management or self-defence techniques. Four studies included not only an educational component, but also organisational and environmental interventions in the ED. Most of the studies observed a positive impact of their intervention on the frequency of violent incidents or the preparedness of ED staff to deal with violent situations. However, due to the limited number of studies, heterogeneity of methods and the limited methodological quality of studies, this result will need to be verified in future research.

In their review of the literature published between the years 1986 and 2007 on interventions to reduce workplace violence against ED nurses, Anderson et al. [37] saw a paucity of research evaluating such interventions and formulated a strong need for further investigations. As shown by the present review, only 15 studies were identifiable in this regard since 2010. Of them, only two were assessed as high-quality studies with a low risk of bias.

Most studies examined behavioural interventions, more precisely training and education. Of them, two studies found no [24] or only very limited evidence [32] for a positive effect of their intervention on the confidence and attitudes of staff regarding de-escalating and managing aggression. Both had examined short (5 and 45-min) in-service training sessions. It could be concluded that longer training sessions are required to achieve a positive impact on staff confidence. Positive effects of short training sessions (e.g., a 10 min video podcast) were observed for knowledge attainment through a test score [23]; however, this does not imply that staff could also handle an aggressive situation better or feel well prepared to do so. In addition, it could be useful to implement frequent and regular repetitions of training. Gillam [25] recommended biannual training, as she observed a decrease of violent events only when more staff had been trained in the previous 90–150 days and not for longer time periods before that. Concerning the form of educational training, it can be noted that online, classroom as well as hybrid programmes were identified in this review, and positive effects were observed by studies for all three forms. In general, online programmes have the advantage of being more flexible in terms of time and pace. On the other hand, classroom programmes allow for the application of interactive exercises, which can make learning, e.g., of de-escalation or self-defence techniques, more effective [38].

Anderson et al. [37] already noted that organisational and environmental problems in EDs cannot be resolved through training interventions. In Germany, it is legally required that occupational health and safety measures at a behavioural level are subordinate to measures at an environmental and organisational level (§ 4 ArbSchG). Guidelines on violence prevention in healthcare as well as recent studies developing frameworks for occupational violence based on the experiences of ED staff, also recommend comprehensive multidimensional approaches [4,39,40]. Three such comprehensive approaches have been identified through this review, all of which showed positive effects on the frequency of violent incidents [14,30,35], but not an advantage over the external comparison group [14]. Similarly, a former systematic review found preliminary evidence for environmental modifications (e.g., specialised behavioural rooms and security upgrades) for acute behavioural disturbance management in EDs, but no evidence from controlled studies [41]. In this review, environmental and organisational preventive measures included, among other things, the implementation of signage, policies and procedures, video surveillance and screening for patients’ risk for violence. The use of risk assessment tools for screening patients was also suggested by ED staff in a survey on interventions for occupational violence [39]. In an Australian ED, the implementation of such a tool to identify ‘at risk’ patients, including a response framework, significantly reduced unplanned violence-related security responses [42]. Cabilan et al. [39] developed a framework for planning occupational violence strategies. As environmental prevention strategies, they recommended, to provide a security presence and duress alarms, to improve staffing and to limit the number of visitors.

### 4.1. Strengths and Limitations

This systematic review included only research articles and no specific search was conducted to identify grey literature, so it is possible that some studies might have been missed. Apart from this, the review provides a comprehensive picture of current workplace violence prevention interventions in EDs through extensive database searching and the inclusion of different study languages. When interpreting the results and conclusions, it should be considered that studies with low quality scores were not excluded from this review. Some of the included studies had shortcomings, e.g., by failing to include an external control group. Most studies did not include multiple measurements after the intervention, preventing conclusions from being drawn about the long-term effects of the interventions. Some had a cross-sectional design and did not provide pre- and post-testing. No conclusions about causal relationships can be derived from these studies. In addition, most studies relied on self-reports of their participants when measuring outcome effects of the intervention, which can increase the risk of bias. Studies included in this review differed considerably in their applied methods, their outcome parameters and the instruments used to measure these. Therefore, it was not possible to pool data, which further limits the results of this systematic review.

### 4.2. Implications

ED staff frequently experience verbal and physical violence from patients and their relatives, which can significantly affect their health. Therefore, workplace violence prevention should be incorporated in emergency clinical care. While staff should be trained in de-escalation and self-defence techniques, organisational and environmental improvements should also be implemented in EDs. A risk assessment, e.g., based on a walk-through, can help to identify specific needs and find appropriate preventive measures for the individual ED [4]. For measures to be successful, it is recommended to involve other stakeholders such as security personnel as well as hospital and ED management to gain leadership support [27]. As shown by this review, preventive measures can have positive effects on the frequency of violent incidents and staff knowledge, skills and confidence to handle critical situations. Implemented interventions should, however, be evaluated for their effectiveness. In this regard, more research is needed to examine the effects of workplace violence prevention interventions. Future studies should take care to use comparable outcome measures, include an external control group and examine long-term effects of interventions by conducting multiple measurements over a longer period of time after the intervention.

## 5. Conclusions

This systematic review provides an overview of current research on workplace violence prevention interventions in hospital EDs. The findings revealed that the included studies mostly showed some positive impact of behavioural and multidimensional interventions on the reduction of violent incidents from patients towards ED staff or the preparedness of staff to deal with violent situations, although the evidence is still sparse. Further studies are needed, that are of high methodological quality and that consider environmental and organisational interventions in particular, as these have rarely been studied so far. This would be an important contribution to promote the introduction of workplace violence prevention interventions in EDs and increase knowledge on the effectiveness of specific measures.

## Figures and Tables

**Table 1:** Eligibility criteria for the screening and selection of studies.

Criteria	Inclusion	Exclusion
Population	Healthcare workers in hospital emergency departments	
Exposure	Violence and aggression by patients and their relatives	Violence due to criminal intent and personal relationship, worker-on-worker violence, use of firearms
Intervention	Prevention or protection approaches in the form of environmental, organisational and behavioural (education and training) interventions	Interventions focusing on documentation, post-incident treatment, pharmacologic sedation or physical immobilisation of patients
Outcome	Frequency of violent incidents, staff knowledge, skills/competencies or awareness, staff sense of well-being and safety	Outcome parameters related to patients
Study design	Interventional studies (e.g., randomised and nonrandomised controlled trials, quasi-experimental studies); observational studies (e.g., cohort studies, cross-sectional studies)	Case studies, reviews
Publication type	Research articles	Letters to the editor/commentaries, conference proceedings, theses and dissertations
Publication date	From 1 January 2010	
Study region	Europe, North America, Australia	Other continents

## Data Availability

No new data were created or analysed in this study. Data sharing is not applicable to this article.

## Supplementary Materials

The following are available online at https://www.mdpi.com/article/10.3390/ijerph18168459/s1, Table S1: Search string for MEDLINE (PubMed), Table S2: Critical appraisal results of the included studies using the JBI Critical Appraisal Checklist for Quasi-Experimental Studies, Table S3: Critical appraisal results of the included studies using the JBI Critical Appraisal Checklist for Prevalence Studies.
