# Supplementary material for: Interventions for Workplace Violence Prevention in Emergency Departments: A Systematic Review
Source: Int J Environ Res Public Health. 2021 Aug 10;18(16):8459. doi: 10.3390/ijerph18168459 (PMC8392011; doi:10.3390/ijerph18168459)
Supplement: Supplementary file 1 [file ijerph-18-08459-s001.zip › Supplementary Table S1.pdf]

Table S1 Search string for MEDLINE (PubMed)

**Population (Occupation)**		**Population (Setting)**		**Exposure**		**Intervention**
Combined with **OR**		combined with **OR**		combined with **OR**		combined with **OR**
health personnel [MeSH]		emergency service, hospital [MeSH]		violen*		intervention*
personnel		emergency department*		WPV		education*
worker*		emergency care		aggression*		inservice training*
staff		emergency nursing		aggressive behavior*		in-service training*
employee*	**AND**	emergency medicine	**AND**	aggressive behaviour*	**AND**	staff development
professional		emergency service*		verbal abuse*		training program*
professionals		emergency unit*		assault*		prevention program*
physician*		emergency room*		harass*		organizational polic*
doctor*		emergency ward*		hostil*		organisational polic*
clinician*						safety management
nurse*						security measure*
nursing assistant*						de-escalat*
caregiver*						
paramedic*						
