# Supplementary material for: Interventions for Workplace Violence Prevention in Emergency Departments: A Systematic Review
Source: Int J Environ Res Public Health. 2021 Aug 10;18(16):8459. doi: 10.3390/ijerph18168459 (PMC8392011; doi:10.3390/ijerph18168459)
Supplement: Supplementary file 1 [file ijerph-18-08459-s001.zip › Supplementary Table S2.pdf]

Table S2 Critical appraisal results of the included studies using the JBI Critical Appraisal Checklist for Quasi-Experimental Studies

**Study**	**Q1:** clarity about 'cause' and 'effect'	**Q2:** similar participants in comparisons	**Q3:** similar treatment/care of participants in comparisons	**Q4:** independent control group	**Q5:** multiple pre/post measure-ments	**Q6:** follow-up complete or described adequately	**Q7:** same way of outcome measurement in comparisons	**Q8:** valid outcome measures[1]	**Q9:** appropriate statistical analysis	**Score**
Ball et al. (2015)	Y	Y	Y	Y	N	Y	U	N	Y	6/9
Buterakos et al. (2020)	Y	Y	Y	N	N	N	Y	N	N	4/9
Gerdtz et al. (2013)	Y	Y	Y	N	N	Y	Y	Y	Y	7/9
Gillam et al. (2014)	Y	N/A	Y	N	Y	N	Y	N	Y	5/9
Gillespie, Farra et al. (2014)	Y	Y	Y	N	Y	N	Y	N	Y	6/9
Gillespie, Gates et al. (2014)	Y	Y	Y	Y	Y	Y	Y	N	Y	8/9
Gillespie et al. (2012)	Y	Y	Y	N	N	U	N	N	Y	4/9
Hills et al. (2010)	Y	N	Y	N	N	N	Y	Y	N	4/9
Krull et al. (2019)	Y	Y	Y	N	N	Y	Y	N	Y	6/9
Okundolor et al. (2020)	U	Y	Y	N	Y	U	U	N	U	3/9
Touzet et al. (2019)	U	N/A	Y	N	Y	N/A	Y	Y	Y	5/9
Wong et al. (2015)	Y	Y	Y	N	N	N	Y	Y	Y	6/9

Abbreviations: Q = Question, Y = yes, N = no, U = unclear, N/A = not applicable; [1] Question 8 was slightly adjusted to determine whether validated instruments were used for the measurement of outcome parameters.
