# Supplementary material for: Interventions for Workplace Violence Prevention in Emergency Departments: A Systematic Review
Source: Int J Environ Res Public Health. 2021 Aug 10;18(16):8459. doi: 10.3390/ijerph18168459 (PMC8392011; doi:10.3390/ijerph18168459)
Supplement: Supplementary file 1 [file ijerph-18-08459-s001.zip › Supplementary Table S3.pdf]

**Table S3** Critical appraisal results of the included studies using the JBI Critical Appraisal Checklist for Prevalence Studies

| **Study** | **Q1:** appropriate sample frame | **Q2:** appropriate recruitment of study participants | **Q3:** adequate sample size | **Q4:** detailed description of study subjects and setting | **Q5:** sufficient coverage of the identified sample | **Q6:** use of valid methods for the identification of the condition | **Q7:** standard and reliable measurement of the condition for all participants | **Q8:** appropriate statistical analysis | **Q9:** adequate response rate | **Score** |
|---|---|---|---|---|---|---|---|---|---|---|
| Bataille et al. (2013) | Y | Y | N | N | N | N | U | N | U | 2/9 |
| Frick et al. (2018) | Y | Y | Y | N | N | N | N | Y | N | 4/9 |
| Gillespie et al. (2013) | U | U | Y | N | N | N | U | N | U | 1/9 |

Abbreviations: Q = Question, Y = yes, N = no, U = unclear, N/A = not applicable
